# ID-GBA: Subgraph Extension With Information Distance Guilt by Association in Complex Networks

**DOI:** 10.1109/access.2025.3622038

**Published:** 2025-10-15

**Authors:** PREDRAG OBRADOVIC, VLADIMIR KOVACEVIC, ALEKSANDAR MILOSAVLJEVIC, VARDUHI PETROSYAN

**Affiliations:** 1 School of Electrical Engineering, University of Belgrade, 11000 Belgrade, Serbia; 2 Persida Inc., Brooklyn, NY 11219, USA; 3 Department of Molecular and Human Genetics, Baylor College of Medicine, Houston, TX 77030, USA; 4 Quantitative and Computational Biosciences Program, Baylor College of Medicine, Houston, TX 77030, USA

**Keywords:** Subgraph extension, biological network, information distance, gene pathways, guilt by association

## Abstract

Here, we introduce the ID-GBA (Information Distance Guilt By Association) method to expand highly connected sets of nodes by deploying a novel algorithm for subgraph extension based on the guilt-by-association principle and information distance. In this study, ID-GBA was utilized to expand disease clusters, and identify novel disease genes. We first validate its ability to expand related disease sets from disease/disease graphs built using Open Targets’ gene association scores. We then analyze disease/control gene expression networks and show that ID-GBA recaptures known disease genes in nine disease/control graphs. Compared to existing methods such as Random Walk with Restarts and Personalized PageRank, ID-GBA achieves significantly higher Normalized Discounted Cumulative Gain scores, which indicates superior predictive performance at capturing known disease genes. Additionally, unlike other approaches that require users to specify either a threshold parameter or a fixed number of nodes to include in the extended subgraph, ID-GBA includes a built-in, automated, and data-driven thresholding mechanism. These results establish ID-GBA as a novel open-source tool to uncover hidden relationships in gene/gene, disease/disease, and other complex networks.

## Graphical Abstract

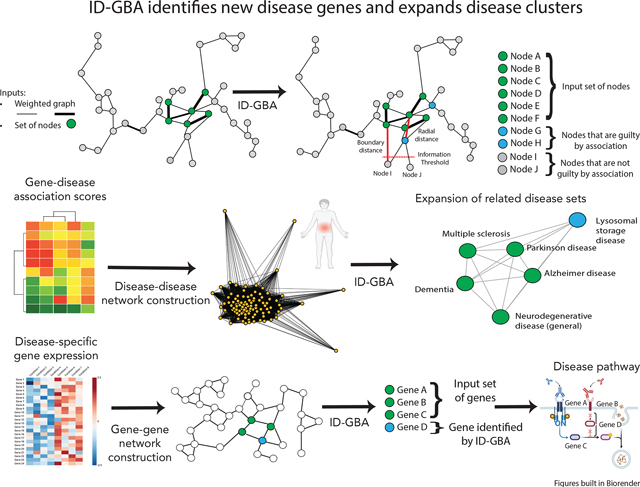

## I. INTRODUCTION

We present ID-GBA, a novel graph-based analysis algorithm that expands an initial marked subset of nodes within a graph to identify new nodes, highly connected to it. Designed to be data-agnostic, it can be applied to any domain where relationships can be represented as a network, including, but not limited to social, knowledge, transportation, and biological networks. As a seed set expansion method, ID-GBA enables the discovery of nodes that are strongly related to a given starting set, supporting hypothesis generation and the identification of novel elements in the network.

In this paper, we demonstrate the algorithm’s applicability in the domain of network biology. For example, in networks connecting diseases that often occur together (disease comorbidity networks), starting from a known disease module ID-GBA can reveal other related diseases, improving understanding of disease relationships and informing treatment strategies. Similarly, in gene co-expression networks, which describe functional relationships between genes, expansion of the initial subset of nodes can identify additional genes that may play important roles in a disease. In both cases, ID-GBA helps uncover previously unrecognized connections, opening new directions for research and discovery.

### A. BACKGROUND AND HISTORY

In our previous work on the Connect the Dots (CTD) algorithm [1], [2], we have addressed the problem of discovering a well-connected subgraph in a set of nodes by using the Algorithmic Significance Theorem [3] to aid in diagnosing patients [4], [5]. However, this work did not address the extension of highly connected subgraphs. The most commonly used method to build and investigate gene-gene co-expression networks is weighted gene co-expression network analysis (WGCNA), which has previously been used to explore co-expression modules to obtain central genes in various diseases such as colorectal cancer, chromophobe renal cell carcinoma, and colon cancer [6], [7], [8], [9]. However, the WGCNA approach relies on eigen genes to identify informative modules, and does not allow for the extension of gene sets. Overall, approaches such as WGCNA either require manually picked thresholds or suffer from high computational demands, motivating the development of ID-GBA.

### B. RECENT FINDINGS

Traditional set extension methods such as shortest paths, random walks, or graph diffusion techniques rely on heuristic-based measures of connectivity [10]. For example, Random Walk with Restarts (RWR) [11] performs a random walk in which each step includes probability of restarting at the source node. Nodes that are frequently visited during the walk receive higher scores, reflecting their proximity to the source. Similarly, Personalized PageRank (PPR) [12] computes a probability distribution over the graph biased toward a set of seed nodes, ranking other nodes based on their relevance to the seeds and capturing local graph structure. Although these algorithms are widely used, they are computationally costly and not well suited to analyzing large or dense disease-disease or gene-gene networks. Additionally, they do not include a principled mechanism for determining how many of the top-ranked nodes should be included in the extended subgraph. Other bioinformatics approaches such as FaBP [10] use more specialized guilt-by-association (GBA) approaches but lack publicly available implementations, making them difficult to reproduce, validate, or apply in practice. Additionally, algorithms such as DREAMwalk [13] are not easily applicable to gene-gene or disease-disease networks. Information theory has successfully been used in algorithm analysis, pseudo random number generation, and feature selection, but also to guide model selection and data fusion strategies in complex biomedical contexts [14]. To address the limitations of existing subgraph extension and GBA algorithms, we developed ID-GBA (Information Distance Guilt By Association). This novel method uses an information-theoretic approach to quantify node distances and propose subgraph extension in an unsupervised manner by providing the ranks of candidate nodes along with an automatic cutoff for the extension process.

## II. RESULTS

### A. ID-GBA EXTENDS DISEASE CLUSTERS FROM GENE ASSOCIATION SCORES

Extending related disease clusters could allow for the identification of shared pathology. To investigate this hypothesis, we built disease/disease association graphs, extended seed disease sets, and counted the scientific papers in which the initial disease of interest is co-mentioned with the diseases in the extended set. To build these graphs we utilized Open Targets [15], which provides gene-disease associations for more than 39,000 gene/disease pairs [15], [16]. The standard Open Target scores are computed from the combination of genetic associations, somatic mutations, RNA expression association, pathway information, drug target information, and existing literature. To allow for the validation of our extended sets, we limited the signal from the literature by removing the literature component of the Open Target scores. These scores were then normalized to a 0–1 scale (0 = no association; 1 = strong association)for each gene/disease association. These scores allowed us to create a disease/disease graph G, where each edge corresponds to the correlation of the diseases by taking into account individual gene association scores from the genes they share. Due to the size and high levels of connectivity in this graph, we pruned it by retaining only the top 5% of edges with the highest weights. To further investigate the disease associations within this graph, we then selected 3 different genetically influenced diseases to focus on: Alzheimer’s disease, childhood cancer, and congenital heart disease. For each disease, we constructed an initial disease association module by including the 20 most genetically correlated diseases [17]. These disease modules were then used as inputs for ID-GBA, along with the general disease/disease graph for set extension. The Entrez PubMed API [18], [19], [20] was then analyzed with Pubtator3 [21] to count the number of articles that mention each of Alzheimer’s disease, childhood cancer, and congenital heart diseases with their extended sets of diseases, respectively.

**FIGURE 1.**
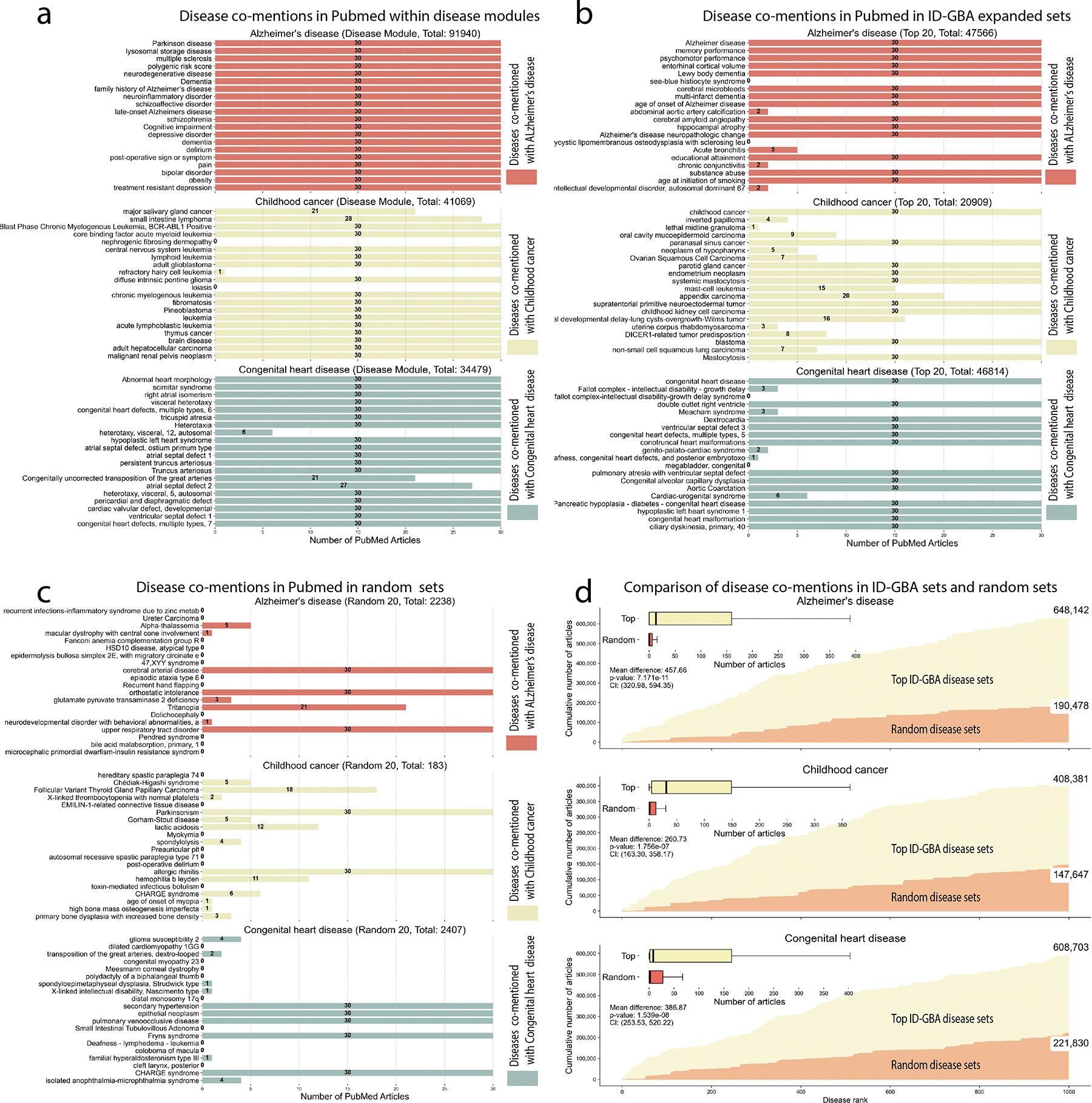
Validation of ID-GBA selection of informative disease subsets. To evaluate the informativeness of disease subsets selected by ID-GBA, we conducted a PubMed co-mention analysis using the PubTator3 package [21]. This analysis quantified how frequently diseases from different sets, including the original disease set, the ID-GBA-expanded set, and randomly generated sets were co-mentioned in the same article as the disease of interest in panels (a), (b), and (c), respectively.(a) For the initial disease sets (e.g., Parkinson’s disease, multiple sclerosis, lysosomal storage disorders), the upper red section shows the number of articles (capped at 30) that mention both Alzheimer’s disease and each disease of interest. The middle yellow and lower blue sections show the same for childhood cancer and congenital heart disease, respectively.(b) For the top ID-GBA expansion sets, the upper red section shows co-mentions with Alzheimer’s disease, and the middle and lower sections correspond to childhood cancer and congenital heart disease, respectively.(c) For random sets of 20 diseases, the same layout and color coding are used as in (a) and (b).(d) This figure compares the number of articles mentioning both the starting disease and each disease from the top 1,000 ID-GBA extensions (yellow) versus a random selection of 1,000 diseases (red). The x-axis shows the rank of ID-GBA extensions, and the y-axis shows the cumulative number of co-mentioning articles, demonstrating a steeper growth for ID-GBA. Box plots below summarize the distribution of article counts for each starting disease. Total article counts are shown on the right side of the panel.

Figure 1a presents the number of articles (capped at 30 for increased legibility) that mention Alzheimer’s disease alongside the 20 diseases from the original disease module (such as Parkinson’s disease, multiple sclerosis, and lysosomal storage disease). This provides a positive baseline for the ID-GBA analysis. The co-mention counts are displayed in the upper red section of the figure, while the middle and lower sections show the corresponding counts for childhood cancer and congenital heart disease, respectively.

Figure 1b follows the same methodology but focuses on the top 20 ID-GBA extensions of the disease module, to determine if the ID-GBA method enriches for known disease-disease associations in the literature. Like the disease set analysis, the upper red section represents the number of articles (limited to 30) that mention Alzheimer’s disease together with these extended diseases (e.g., glioblastoma multiforme, lung adenocarcinoma, and neoplasm). The middle and lower sections depict the same associations for childhood cancer and congenital heart disease, respectively. Finally, we perform the same analysis with 20 randomly selected diseases in Figure 1c, to provide a negative control for the ID-GBA analysis. As shown in the bar chart, the ID-GBA extensions demonstrate a stronger association with the initial diseases than the random set of diseases. For example, for Alzheimer’s disease, 18/20 of the diseases in the ID-GBA expanded set had at least 2 co-mentions with Alzheimer’s disease in the literature. Likewise, the ID-GBA expansion of childhood cancer and congenital heart disease had at least 2 co-mentions with the disease of interest for 19 and 17 diseases in the expanded set, respectively. In contrast, the randomly selected diseases exhibit much weaker associations, with only 6, 11, and 8 of the random sets having at least 2 co-mentions for these respective diseases. This confirms that the extension of the disease set proposed by ID-GBA has significantly higher relevance and confidence than expected by random selection. This result also supports the hypothesis that the ID-GBA extension identifies diseases that have more shared pathology than by chance, and allows us to hypothesize that the diseases in the ID-GBA sets that are not currently co-mentioned in the literature may also share common pathologies.

To further investigate the significance of the disease module on a broader scale, we compared the number of articles that mention both the initial disease (Alzheimer’s disease, childhood cancer, or congenital heart disease) and each disease of the top 1,000 ID-GBA extensions (yellow) with those from a randomly selected set of 1,000 diseases (red), as shown in Figure 1d. The x-axis represents the rank of the disease extensions proposed by ID-GBA or by random selection, while the y-axis shows the cumulative number of articles mentioning each disease in combination with the corresponding starting disease. For all three selected diseases, the distribution of article counts for both the top 1,000 ID-GBA extensions and the randomly selected 1,000 diseases is visualized through box plots below. Statistical analysis shows a significant difference between the two groups, and that the ID-GBA extensions have statistically more co-mentions than the random subsets.

- **Alzheimer’s disease**: Mean difference of 457.66 articles, p-value = 7.171e-11, confidence interval (320.98, 594.35).
- **Childhood cancer**: Mean difference of 260.73 articles, p-value = 1.756e-07, confidence interval (163.30, 358.17).
- **Congenital heart disease**: Mean difference of 386.87 articles, p-value = 1.539e-08, confidence interval (253.53, 520.22).

The total number of articles found for each disease in the top 1,000 ID-GBA extensions versus the randomly selected 1,000 diseases is summarized on the right side of Figure 1d:

- **Alzheimer’s disease**: 648,142 articles for the top 1,000 ID-GBA extensions vs. 190,478 for the random 1,000 diseases.
- **Childhood cancer**: 408,381 articles for the top 1,000 ID-GBA extensions vs. 147,647 for the random 1,000 diseases.
- **Congenital heart disease**: 608,703 articles for the top 1,000 ID-GBA extensions vs. 221,830 for the random 1,000 diseases.

### B. ID-GBA RECAPTURES KNOWN DISEASE GENES AND IDENTIFIES DISEASE RELEVANT PATHWAYS

**FIGURE 2.**
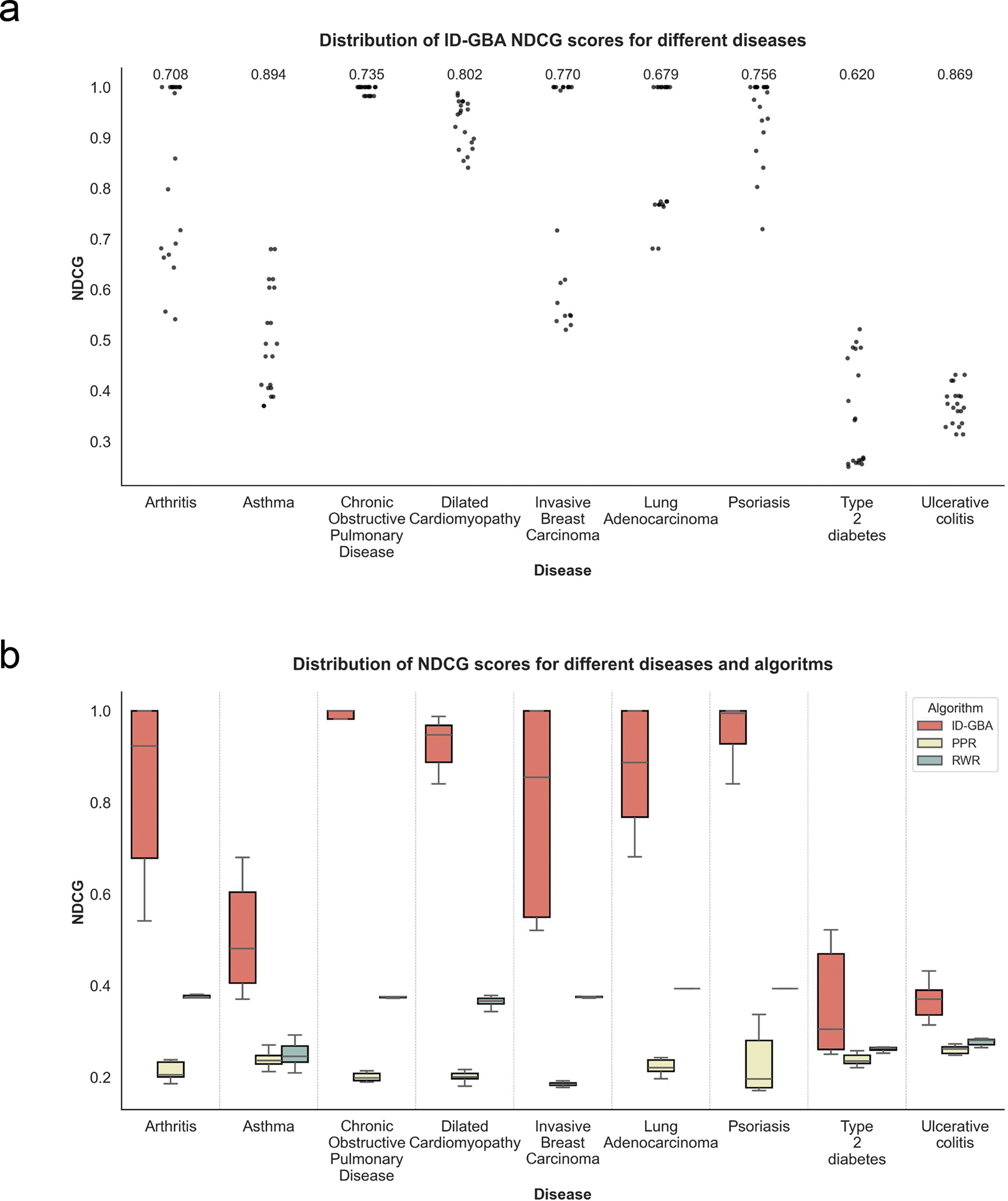
Distribution of NDCG scores for gene expression graphs of 9 diseases. a) ID-GBA’s NDCG scores for predicting ranks for subgraph extension of 10 different 80:20 subgraph splits. Nine conductance scores for each disease subgraph are given on the top part of the diagram b) Comparison of NDCG scores for predicting ranks of remaining 20% of the nodes of selected subgraph obtained with ID-GBA, PPR, and RWR algorithms.

To identify genes associated with disease and capture disease pathology we built disease/control gene co-expression networks for 9 distinct diseases. We selected the subgraphs of disease relevant genes, as described in Methods, by performing unsupervised spectral clustering and selecting a cluster with the smallest conductance (Supplementary Fig. 2 available in the Supplementary Results). To determine if ID-GBA extension can recapture known disease genes, we performed 10 different random 80–20 splits and ran the ID-GBA algorithm on 80% of the nodes to propose the scores for extension of the remaining 20%. To measure the scores of the extended gene set, we utilized Normalized Discounted Cumulative Gain (NDCG) [22] scaled from 0 to 1, where 1 corresponds to assigning the top ranks to the remaining 20% of nodes in this case. Figure 2a shows the distribution of NDCG scores per disease graph for the different 80–20 splits. High NDCG scores seen in Figure 2a indicate good detection capabilities of the remaining 20% of nodes that belong to the selected subgraph cluster. For each disease extension, we also determined the subgraph conductance to measure the information flow within the graph. For each disease extension set, a small conductance value corresponds to more potential for extension.

As a part of the statistical analysis, we computed the mean NDCG score, standard deviation, 95% confidence intervals, and *p*-values for each disease network when using the ID-GBA algorithm given in the Supplementary Table 1. The results show that for the majority of disease networks, the NDCG scores are consistently high, with relatively narrow confidence intervals, indicating stable performance. Statistically significant differences (i.e., *p* < 0.05) were observed in several networks, demonstrating that the improvements of ID-GBA over baseline methods are not limited to a single case. These results strengthen our claim that the performance gain of ID-GBA is robust across diverse disease contexts while significantly outperforming the existing methods.

We then investigated if the genes in the disease informative clusters were enriched for disease specific pathways using KEGG [23], [24], [25]. Over representation analysis through the clusterProfiler’s enrichKEGG function discovered the pathways shown in Table 1. With the KEGG analysis, we identified pathways that are known to be associated with our diseases of interest. These include the motor protein pathway in arthritis, the spliceosome pathway that plays a role in the progression of invasive breast cancer, the PI3K-Akt which regulates progression and lung adenocarcinoma, and the FoxO pathway which regulates Tregs and has been associated with psoriasis. The thermogenesis pathway plays a role in managing type 2 diabetes. Additionally, olfactory transduction is associated with ulcerative colitis although its role has not been well studied. There were no significantly enriched pathways for the clusters of the chronic obstructive pulmonary disease or dilated cardiomyopathy graphs, but this may have been due to the low number of genes within these clusters (n = 13 for both graphs). Through this analysis we show that ID-GBA recaptures disease genes within disease gene modules, and that its extended sets are enriched for disease-relevant pathways.

### C. COMPARISON WITH THE EXISTING SOLUTIONS

To further evaluate the performance of ID-GBA, we then compared its performance to other GBA extension methods including RWR and PPR for all 9 disease/control graphs. Figure 2b shows the distribution of NDCG scores of ID-GBA, RRW and PPR from gene expression graphs in the 9 different diseases/control graphs that were used in the prior analysis. Higher NDCG scores of ID-GBA on each disease graph confirms that it has better precision in capturing genes relevant to the disease, and thus, larger subgraph extension potential.

### D. EXECUTION TIME

The execution times for ID-GBA analyses varied primarily depending on the size of the input network, and secondarily on the number of nodes in the disease module, as shown in Table 2. For the smaller 9 graphs comprising approximately 2,000 nodes, the runtime was consistently around 2 to 3 seconds across different diseases, reflecting efficient computational performance. For larger 3 networks used for gene-disease associations that contain over 13,000 nodes, the execution times were longer, approximately 60 to 65 seconds. This increase corresponds with the greater complexity and computational demands associated with larger adjacency matrices. Overall, the results demonstrate that the method scales predictably with network size while maintaining practical runtime for typical disease modules.

## III. METHODS

### A. OVERVIEW OF INFORMATION DISTANCE

Information distance, as defined in computer science [26], has previously been used to measure the similarity or distance of strings, sequences, or objects utilizing the concept of Kolmogorov complexity. However, in the context of this research, we use the term “information distance” to represent the distance between two nodes in a network, modeling the ease of information propagation between them. In our definition of information distance between two nodes, fewer bits would correspond to a shorter distance and better connectivity. Likewise, more informational bits needed to encode node B starting from node A would correspond to a larger distance and weaker connection, possibly signifying randomness and absence of meaningful connectivity patterns between the nodes. Although this is different from the standard computer science definition of information distance, we believe that the difference in domains will ensure that there is a minimal chance of confounding the two terms.

**Algorithm 1:** Thresholded Information Distance

**Require:** Weighted network G=(V,E) with weight matrix $W=\left(w_{i,j}\right)$ and a seed node set S	
**Ensure:** Thresholded information distance ${id}_{t}(i,j)$ for all i∈S,j∈V	
1:	**Compute node degrees:**
	$deg(i)\leftarrow \sum_{k\in V}w_{i,k},\;\forall i\in V$
2:	**Row-normalize adjacency matrix:**
	$p_{i,j}\leftarrow \frac{w_{i,j}}{deg(i)},\;\forall (i,j)\in E$
	This gives the probability of moving from i to j in one step.
3:	**Compute information hop distance matrix (IHD):**
	$ihd(i,j)\leftarrow -log\;p_{i,j},\;\forall (i,j)\in E$
	This corresponds to self-information of the transition.
4:	**Compute information distance matrix (ID):**
	For each node pair (i,j), where i∈S run a shortest-path algorithm (e.g., modified Dijkstra) on the information hop distance matrix to find:
	$id(i,j)\leftarrow \underset{\begin{array}{c} \left(v_{0},v_{1},\ldots ,v_{k}\right)\in 𝒫(G)\\ v_{0}=i,v_{k}=j \end{array}}{min}\;\sum_{\ell =0}^{k-1}ihd\left(v_{\ell },v_{\ell +1}\right)$
	where 𝒫(G) is the set of all paths in the graph.
5:	**Compute equal-length encoding threshold:**
	$T\leftarrow {log}_{2}(|V|)$
	This is the number of bits needed to encode any node under the null model of random connectivity.
6:	**Apply threshold to obtain thresholded information distance:**
	${id}_{t}(i,j)\leftarrow min(id(i,j),T)$
7:	**return** ${id}_{t}(i,j)$ for all i∈S,j∈V

The concept of information distance emerges naturally if we try to measure the ease of passing information between two nodes A and B in a weighted network using information-theoretic terms. To determine the information distance between two nodes, we measure the least number of bits needed to iteratively encode node B starting from node A, drawing inspiration from the probabilistic encoding scheme with a Markov walker, employed by the CTD encoding algorithm [4]. Then, we form a null hypothesis about absence of connectivity in the graph and compute a distance threshold from this baseline model for equal length encodings. Finally, we apply this threshold to obtain thresholded information distance. The algorithm for computation of thresholded information distance is shown in Alg. 1, with the following subsections explaining it in detail.

### B. TRANSITION PROBABILITIES

To model information flow between nodes, we create a row-normalized adjacency matrix P. This is done by dividing each edge’s weight by the total weight of all outgoing edges from the source node (node’s out-degree). This normalization ensures that the weights in each row sum to 1, reflecting the relative strength of connections from that node. The row-normalized adjacency matrix is the transition probability matrix of our network, where each element $p_{i,j}$ corresponds to the probability of direct transition from node i to node j.

### C. INFORMATION HOP DISTANCE

The row-normalized adjacency matrix models the probability of transition between nodes in the network. In order to transform the transition probabilities into distances between neighboring nodes (which we name information hop distances), we employ Shannon’s concept of self-information to encode transitions in the graph, attributing shorter codes (and thus lower distances) to transitions that happen more often. Therefore, information hop distance between two neighboring nodes i and j connected by an edge $e_{i,j}$ with weight $w_{i,j}$ is calculated as

(1) $$ihd(i,j)=-log\frac{w_{i,j}}{deg(i)}=-log\frac{w_{i,j}}{\sum_{k}\;w_{i,k}}=-log\;p_{i,j}$$

Using the previously obtained transition matrix P and applying the negative logarithm element-wise, we obtain the information hop distance matrix IHD:

(2) IHD=−log(P).

Therefore, information hop distance is the self-information of the direct transition between neighboring nodes. The logarithmic transformation ensures a distance-like property, which can be used to identify close neighbors in a network.

### D. OBTAINING INFORMATION DISTANCES

In order to calculate the information distance between two nodes that are not neighbors, we use the information hop distance matrix IHD which contains the distances between neighbors and apply an algorithm for finding the shortest path between nodes. The shortest path between two nodes in this “information space” is called *information distance*. If we denote the set of all paths in G with 𝒫(G), the information distance is computed as

(3) $$id(i,j)\leftarrow \underset{\begin{array}{c} \left(v_{0},v_{1},\ldots ,v_{k}\right)\in 𝒫(G)\\ v_{0}=i,v_{k}=j \end{array}}{min}\;\sum_{\ell =0}^{k-1}ihd\left(v_{\ell },v_{\ell +1}\right)$$

In ID-GBA, we perform this step with the modified Dijkstra algorithm using Fibonacci heaps from the SciPy Python package [27], because it is already parallelized and optimized to efficiently compute distances from a specific set of source nodes, instead of calculating distances between every possible pair of nodes. This is important for use in the ID-GBA algorithm, which needs to compute information distances from nodes in the seed set S to all nodes in G.

### E. EQUAL-LENGTH ENCODING THRESHOLD AND THRESHOLDED INFORMATION DISTANCE

In many recommender systems, the number of items to recommend is fixed in advance by specifying a *top-k* parameter. This approach is simple and widely used in methods based on Personalized PageRank [28] and Random Walk with Restart [29]. However, it imposes an arbitrary cutoff that can lead to two key drawbacks: (i) omitting items that, although ranked below the cutoff, remain highly relevant, and (ii) including marginally relevant items solely to meet the quota. This rigid list-size constraint ignores variations in the strength of connections between items and often requires manual tuning of *k* for each dataset or application context. The PerK framework [30] seeks to address this by personalizing the recommendation list size, but it is not directly applicable to graph data and incurs substantial computational overhead. In contrast, our approach removes the need for this parameter entirely by introducing a data-driven threshold that automatically determines which recommendations are significant. This threshold, derived from the interpretation of the information distance under a null model of random connectivity, requires no additional computational cost.

To determine which information distances should be considered close or far, we first require a baseline for comparison. We establish this baseline by adopting the null hypothesis that the graph has no underlying structural connectivity or hidden patterns, meaning that all connections are purely random. Under this assumption, the optimal encoding scheme is an *equal-length encoding scheme*, in which nodes are ordered and each is encoded using exactly ${log}_{2}(|V|)$ bits.

This implies that, starting from any node *A* in the graph, we can encode any other node *B* using exactly ${log}_{2}(|V|)$ bits, simply by applying the equal-length encoding scheme to node *B*. Importantly, no prior knowledge is considered for node A, that would reduce the encoding cost of node *B*, because in the case of the null hypothesis, we cannot assume any structural regularity or patterns in the graph.

Equal-length encoding with the null hypothesis is then used to calculate the **threshold value**. If the information distance between two nodes *A* and *B* exceeds ${log}_{2}(|V|)$, then the encoding scheme based on network structure performs worse than the naive equal-length encoding. In this case, the nodes are considered *less connected* than a pair of randomly selected nodes under the null hypothesis. Conversely, if their information distance is smaller than ${log}_{2}(|V|)$, the nodes are *better connected* than expected under randomness, and thus have a significant connection. This leads us to define a *thresholded information distance*, which is denoted ${id}_{t}(i,j)$. Thresholded information distance caps all distances at the threshold set by the equal-length encoding scheme:

(4) $${id}_{t}(i,j)=min\left(id(i,j),{log}_{2}(|V|)\right)$$

This built-in thresholding mechanism allows us to identify and focus on connections that are stronger and more significant than expected under the null model of random connectivity. The thresholded information distance corresponds to the minimum number of bits required to encode node *B* starting from node *A*, using either the probabilistic encoder described in [4] or the equal-length encoding scheme, depending on which yields a shorter encoding for the specific node pair.

### F. GUILT BY ASSOCIATION ALGORITHM BASED ON INFORMATION DISTANCE

Our algorithm applies the GBA principle to the problem of recommending an extension of a subgraph. It uses an information-theoretic approach and the thresholded information distance to rank nodes and their relevance for inclusion in the extension. Below, we detail the construction and evaluation steps of the algorithm.

The node ranking is based on the minimum thresholded information distance from one of the key points of the subgraph. These key points are determined based on which distance metric we use.

1. **Radial Distance Metric:** *The center of the subgraph* is identified by finding the node or set of nodes with minimal eccentricity. Node eccentricity is defined as the maximum shortest path distance from the node to any other node in the subgraph. When using the radial distance metric, the center of the subgraph is used as the set of key points.
2. **Boundary Distance Metric:** *The inner boundary* is identified as a set of nodes within the subgraph that have at least one neighbor outside the subgraph. These nodes provide a natural demarcation for extending the subgraph and are used as key points for the boundary distance metric.

For a given set of nodes S (subgraph), we calculate the pairwise thresholded information distances within S and from S to all other nodes in the graph using Dijkstra’s algorithm. The resulting distance matrix is used for both radial and boundary metrics. Nodes not in S are ranked by their thresholded information distance from the key points (centers or boundary nodes), facilitating their evaluation for potential inclusion in the extension of the subgraph. The distance metric determines how nodes are evaluated and ranked as potential candidates for extending the subset S.

The use of thresholded information distance removes the need to arbitrarily select a cutoff point for subgraph extension, which is a common limitation of other GBA algorithms. In ID-GBA, the cutoff is applied to the ranked recommendation list once the equal-length encoding threshold is reached. All nodes ranked below this point are considered less connected than expected under the null model and are therefore excluded as candidates for subgraph extension. This automated threshold selection allows for the generation of data driven extensions. The ID-GBA ranking process produces a ranked list of nodes, which are ordered by their relevance to the subgraph based on the chosen distance metric. Therefore, ID-GBA represents a recommender system for subgraph extension based on the guilty-by-association principle with a built-in threshold for extension termination.

#### 1) CONDUCTANCE EVALUATION

Subgraph conductance, a well-known network-theoretic measure, is employed to evaluate the information coherence and robustness of the subgraph and justify the GBA-based extension of a subgraph. A subgraph with a high conductance means that a large portion of information is exiting the subgraph. This makes it difficult to employ GBA-based reasoning to expand the subgraph. A low subgraph conductance signifies that the subgraph is well isolated from the remainder of the graph and that it is well clustered. Therefore, we can employ the GBA reasoning principle to expand the subgraph.

Subgraph conductance of a subgraph S is defined as:

$$\Phi (S)=\frac{Cut(S,\;\overset{‾}{S})}{min(Vol(S),\;Vol(\overset{‾}{S}))}=\frac{\sum_{i\in S,\;j\in \overset{‾}{S}}a_{ij}}{min\left(\sum_{i\in S}a_{ij},\;\sum_{i\in \overset{‾}{S}}a_{ij}\right)}$$

where $Cut(S,\overset{‾}{S})$ is the sum of the edge weights connecting S to its complement $\overset{‾}{S}$, and Vol(A) is the total edge weight from set A. Low conductance indicates a well-separated subgraph, suggesting a natural cluster, whereas high conductance implies weak modularity, which would mean that further analysis is needed before the set can be expanded.

It is important to note that while low subgraph conductance correlates with better GBA results, the reverse is not necessarily true. This can be illustrated by imagining S as a subset of a cluster C in G. In such a case, the subgraph conductance of S may be high due to the large number of edges within C. However, since S is a subset of C, there may still be value in using GBA-based reasoning to reconstruct C starting from S.

#### 2) IMPLEMENTATION AND COMPUTATIONAL DETAILS

The ID-GBA algorithm is implemented in Python programming language and utilizes commonly used Python libraries including numpy, pandas, and scipy.

### G. CONSTRUCTION OF GENE CO-EXPRESSION GRAPHS AND SUBGRAPH SELECTION FOR DISEASE COMMUNITIES

To determine the utility of ID-GBA in analyzing gene expression data, we created 9 disease/control gene co-expression networks.

The diseases and their corresponding datasets are detailed in Table 1. For each disease the following analysis was performed to generate a graph and identify clusters:

1. Gene expression profiles were obtained in tabular format, with genes as rows and samples as columns. This data was transposed and z-score normalized.
2. The variance of each gene across all samples was computed. The top 2000 most variable genes were selected for downstream analysis to focus on highly variable genes. Thus, the obtained disease graphs contain 2000 nodes and they are fully connected (2 million branches).
3. Covariance matrices were computed separately for case (disease) and control samples. Precision matrices were calculated by matrix inversion or, if necessary, regularized using EmpiricalCovariance from scikit-learn to handle singular matrices. A differential co-expression matrix was generated by subtracting the control precision matrix from the combined case-control precision matrix, and the result was symmetrized to ensure it could serve as an adjacency matrix for graph construction.
4. Spectral clustering was applied to the differential co-expression matrix, treating it as a weighted adjacency matrix. The number of clusters was set to 40, and genes were assigned to clusters based on the graph structure.
5. Dimensionality reduction was performed using the Uniform Manifold Approximation and Projection (UMAP) algorithm to project the graph into two dimensions. Scatterplots were created to visualize the spatial distribution of genes and their assigned clusters in the UMAP space.
6. Each cluster was evaluated using conductance, a measure of intra-cluster connectivity relative to inter-cluster separation. The cluster with the lowest conductance, representing the most tightly connected subgraph, was selected for further analysis.
7. For each disease, the adjacency matrix of the differential co-expression graph and the gene list of the selected cluster were saved as CSV files. Validation of the results was performed using an external script (test_gba.py), and outputs were logged for reproducibility.

This pipeline enables the systematic identification of disease-specific gene communities and provides insights into underlying molecular mechanisms. This methodology is applicable for finding a highly connected community within the graph, but it does not guarantee the cluster’s optimality. However, this analysis fulfills the purpose of identifying a starting subgraph S for testing GBA extension algorithms.

There are several restrictions on the size of clusters, which will impact the choice of clustering algorithm used to obtain possible starting modules S. Our goal is to create recommendations for the starting module S that partially or fully overlap with some known biological pathways. In addition, the recommended S module needs to be small enough to be interpretable for the medical professional using ID-GBA. Finally, the module with low conductance gives more robust recommendations, as it corresponds to stronger guilt-by-association.

Applying Louvain [31] or Leiden [32] clustering algorithms or other self-tuning resolution-based methods [33], where the number of clusters does not need to be specified in advance, is desirable. However, Leiden and Louvan algorithms and other modularity-based approaches struggle with finding small clusters, because the modularity of partitions including several small clusters is typically worse, even though the cut score could be better. Therefore, we chose to use spectral clustering [34], [35] to enforce the existence of smaller clusters that are easier to interpret. The existence of smaller clusters can be achieved by carefully choosing *k*, the parameter of spectral clustering corresponding to the wanted number of clusters. For bulk RNA-seq data, we expected 10–20 clusters, corresponding to major functional modules (e.g., metabolism, immune response, cell cycle, etc). However, we selected smaller clusters that could be used as the starting module *S* which were relatively comparable to the size of biological pathways and small enough to be easily interpretable. Hufeng et al. [36] reported 42.7 as average number of genes in a pathway, and we used this average number of genes to determine that *k* = 40 was a suitable number of clusters.

## IV. CONCLUSION

This study has demonstrated the applicability of ID-GBA in identifying and extending biologically significant sets of diseases and genes. We show that ID-GBA correctly prioritizes missing members of known sets, particularly in cases where influential nodes were removed. This highlights its robustness in the reconstruction of informative sets. In our first analysis, we build disease/disease graphs and extend disease modules with ID-GBA for Alzheimer’s disease, congenital heart disease, and childhood cancer. We then validate the extended disease sets by demonstrating that the number of PubMed co-mentions between the original disease and its extended set is significantly higher than expected by chance. The extension of disease sets can be used to link diseases with common pathology, explore common disease mechanisms, and find common druggable targets within highly connected sets of diseases.

We then apply ID-GBA to gene co-expression networks constructed for nine diseases, and systematically validate its predictive power. By leveraging an unsupervised spectral clustering approach to extract biologically relevant subgraphs, we demonstrate that ID-GBA effectively identified genes co-regulated within disease-specific pathways. High NDCG scores across the different diseases confirm that the algorithm consistently ranks meaningful genes at the top. This reinforces the utility of information distance metrics in biological inference. Furthermore, the KEGG [23] pathway enrichment analysis showed that clusters identified by ID-GBA correspond to known disease mechanisms, validating the significance of its predictions. The absence of enriched pathways in certain diseases, such as chronic obstructive pulmonary disease and dilated cardiomyopathy, may be due to the limited representation of genes in these groups rather than algorithmic limitations. We then compare ID-GBA to established graph-based inference methods, such as Random Walk with Restarts (RWR) and Personalized PageRank (PPR), and find that it has superior performance across all disease networks. The significantly higher NDCG scores obtained by ID-GBA suggest that its information-theoretic approach provides a more nuanced understanding of node connectivity, facilitating improved subgraph extensions. Unlike RWR and PPR, which rely predominantly on local connectivity patterns, ID-GBA integrates global network topology through information distance, leading to a more comprehensive identification of relevant nodes. In conclusion, ID-GBA presents a powerful framework for extending highly connected sets in complex graphs. The application of ID-GBA to identify significantly connected sets of disease sets the stage for the identification of cross-disease pathology. Furthermore, ID-GBA’s application to gene-gene networks illustrates its potential for identifying novel disease-associated genes and provides a data-driven approach to hypothesis generation in biomedical research. Future studies that apply ID-GBA to multi-omic data and utilize dynamic network analyses could further enhance its applicability in systems biology and beyond.

## Supplementary Material

supplementary_material

## Figures and Tables

**TABLE 1.** Diseases and their associated pathways. The top enriched pathways for each disease are in the table below. These pathways are derived from the genes identified as significant by ID-GBA.

Disease	Associated Pathways
Arthritis	Cytoskeleton in muscle cells, Adrenergic signaling in cardiomyocytes, Cardiac muscle contraction, Hypertrophic cardiomyopathy, Motor proteins
Asthma	Olfactory transduction
Chronic obstructive pulmonary disease	NA
Dilated cardiomyopathy	NA
Invasive breast cancer	Spliceosome
Lung adenocarcinoma	Complement and coagulation cascades, Glycolysis / Gluconeogenesis, Adipocytokine signaling pathway, Insulin resistance, PI3K-Akt signaling pathway
Psoriasis	FoxO signaling pathway
Type 2 diabetes	ATP-dependent chromatin remodeling, Thermogenesis
Ulcerative colitis	Olfactory transduction

**TABLE 2.** Summary of graph sizes, disease module sizes, and execution times for each disease network analyzed.

Disease	Graph size	Disease module size	Execution time (s)

Ulcerative colitis	2000	68	2.46
Invasive Breast Carcinoma	2000	8	1.97
Asthma	2000	63	2.43
Lung Adenocarcinoma	2000	22	2.04
Dilated Cardiomyopathy	2000	22	2.11
Arthritis	2000	66	2.34
Psoriasis	2000	98	2.80
Chronic Obstructive Pulmonary Disease	2000	47	2.20
Type 2 diabetes	2000	84	2.52
Alzheimer’s disease	13283	20	65.00
Childhood cancer	13283	20	61.00
Congenital Heart Disease	13283	20	61.00

## Data Availability

To investigate the specificity of ID-GBA when identifying disease-specific gene sets, we first obtained case/control gene expression data for each disease to build disease-specific graphs. These expression values were obtained from (DiSignAtlas [37] with the following term ids: DSA05072 Ulcerative Colitis (872 cases, 458 controls) DSA08946 Invasive Breast Carcinoma (1103 cases, 112 controls) DSA01344 Asthma (440 cases, 253 controls) DSA08970 Lung Adenocarcinoma (526 cases, 59 controls) DSA08751 Dilated Cardiomyopathy (163 cases, 155 controls) DSA07529 Arthritis (150 cases, 28 controls) DSA07139 Psoriasis (89 cases, 80 controls) DSA09769 Chronic Obstructive Pulmonary Disease (98 cases, 64 controls) DSA00763 Type 2 Diabetes (12 cases, 2 controls) Disease gene associations are downloaded from Open Targets database, data version 25.03. The code for downloading and preparing the data is available in Jupyter notebook.

## CODE AVAILABILITY

ID-GBA is implemented in Python as part of the Connect The Dots (CTD) framework available in Github repository. For comparison with the existing methods, the implementations of PPR and RWR were taken from the Python module pyrwr, available on the corresponding Github repository. Initial analysis and testing of the ID-GBA were performed on the Cancer Genomics Cloud platform [38]. The code for generating figures of this manuscript is given in the Python Jupyter notebooks.
