# Supplementary material for: ID-GBA: Subgraph Extension With Information Distance Guilt by Association in Complex Networks
Source: IEEE Access. Author manuscript; Available in PMC 2026 Mar 10. (PMC12970960; doi:10.1109/access.2025.3622038)
Supplement: supplementary_material [file NIHMS2139272-supplement-supplementary_material.pdf]

Supplementary

February 14, 2026

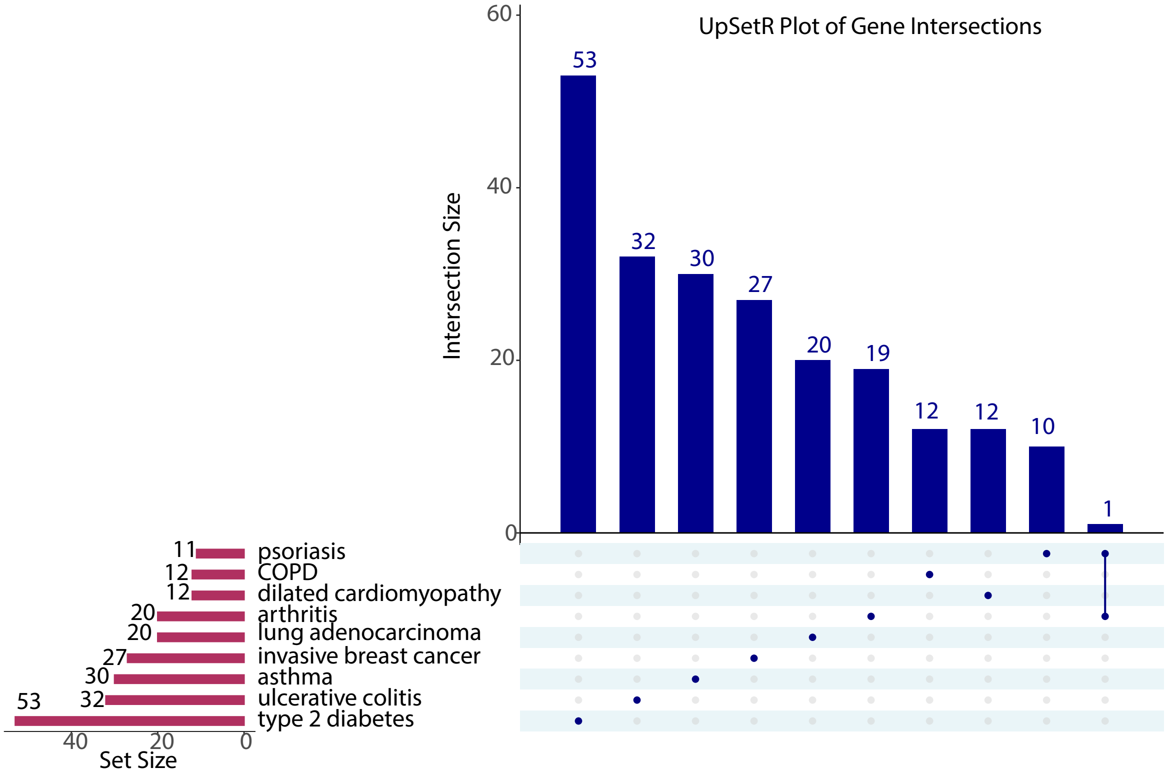

Supplementary Fig. 1: Gene intersections between nine diseases processed with ID-GBA, visualized using an UpSetR plot. Each horizontal bar on the left shows the total number of genes in the representative cluster for a disease. The vertical bars in the main panel show how many genes are either unique to a disease (single black dot in the lower matrix) or shared between diseases (connected black dots). Numbers above vertical bars indicate the gene counts for each category. Most identified genes are unique to a single disease cluster, with the exception of MIR208B, which appears in both psoriasis and arthritis clusters.

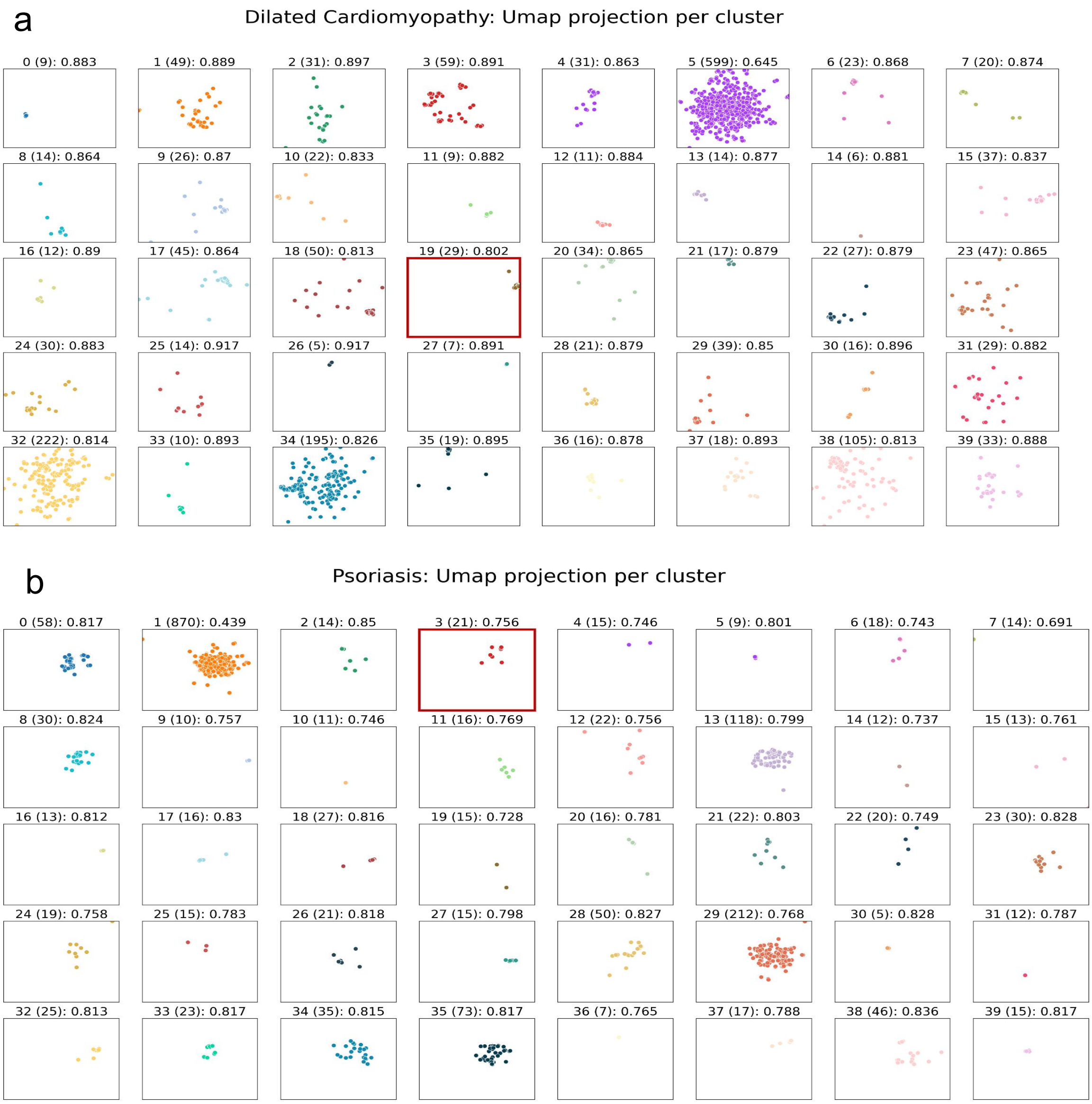

Supplementary Fig. 2: UMAP projection of clusters obtain with unsupervised spectral clustering for a) Chronic obstructive pulmonary disease and b) psoriasis. The cluster with smallest conductance is marked with red rectangle.

Table 1: **Mean NDCG scores, variability, 95% confidence intervals, and p-values for ID-GBA comparisons across diseases.**

Disease	Algorithm	Mean	SD	n	CI Lower	CI Upper	p-value
Arthritis	PPR	0.213	0.020	10	0.199	0.227	4.44×10^{-8}
Arthritis	RWR	0.373	0.007	10	0.368	0.378	3.01×10^{-6}
Asthma	PPR	0.239	0.017	10	0.226	0.251	2.97×10^{-5}
Asthma	RWR	0.248	0.029	10	0.228	0.269	3.17×10^{-5}
Chronic Obstructive Pulmonary Disease	PPR	0.200	0.009	10	0.194	0.206	1.32×10^{-31}
Chronic Obstructive Pulmonary Disease	RWR	0.374	0.001	10	0.373	0.375	6.20×10^{-19}
Dilated Cardiomyopathy	PPR	0.201	0.011	10	0.193	0.209	2.88×10^{-12}
Dilated Cardiomyopathy	RWR	0.363	0.013	10	0.353	0.373	1.87×10^{-11}
Invasive Breast Carcinoma	PPR	0.184	0.006	10	0.180	0.188	5.35×10^{-9}
Invasive Breast Carcinoma	RWR	0.375	0.001	10	0.374	0.376	2.11×10^{-6}
Lung Adenocarcinoma	PPR	0.222	0.016	10	0.210	0.233	1.55×10^{-14}
Lung Adenocarcinoma	RWR	0.393	0.000	10	0.392	0.393	2.34×10^{-10}
Psoriasis	PPR	0.226	0.066	10	0.178	0.273	4.88×10^{-13}
Psoriasis	RWR	0.393	0.000	10	0.393	0.393	1.81×10^{-8}
Type 2 Diabetes	PPR	0.238	0.013	10	0.228	0.247	3.49×10^{-4}
Type 2 Diabetes	RWR	0.261	0.004	10	0.258	0.265	0.390
Ulcerative Colitis	PPR	0.259	0.009	10	0.253	0.266	4.64×10^{-6}
Ulcerative Colitis	RWR	0.279	0.011	10	0.271	0.287	2.00×10^{-5}
